# The Study of Physico-Mechanical Properties of Polylactide Composites with Different Level of Infill Produced by the FDM Method

**DOI:** 10.3390/polym12123056

**Published:** 2020-12-20

**Authors:** Anna Gaweł, Stanisław Kuciel

**Affiliations:** Faculty of Materials Engineering and Physics, Institute of Materials Engineering, Tadeusz Kosciuszko Cracow University of Technology, Al. Jana Pawła II 37, 31-864 Cracow, Poland; zajac.zajacanna@gmail.com

**Keywords:** 3D printing, polylactic acid, FDM method, crystallization, hydrolytic degradation, different degrees of infill, Vicat temperature, hysteresis loops

## Abstract

The aim of this study was to evaluate the changes in physical-mechanical properties of the samples manufactured by 3D printing technology with the addition of varying degrees of polylactide (PLA) infill (50, 70, 85 and 100%). Half of the samples were soaked in physiological saline. The material used for the study was neat PLA, which was examined in terms of hydrolytic degradation, crystallization, mechanical strength, variability of properties at elevated temperatures, and dissipation of mechanical energy depending on the performed treatment. A significant impact of the amount of infill on changeable mechanical properties, such as hydrolytic degradation and crystallization was observed. The FDM printing method allows for waste–free production of light weight unit products with constant specyfic strength.

## 1. Introduction

Three-dimensional (3D) printing, commonly known as additive manufacturing, has recently become a very popular way for rapid manufacturing of various objects in applied chemistry, biomedicine, material science and many other branches of research and industry [1]. This technique enables the manufacture of various functional components or prototypes, even those with complex geometry. Three-dimensional printing using such processes as fused deposition modelling (FDM), stereolithography apparatus (SLA), continuous liquid interface production (CLIP), digital light processing (DLP) and selective laser sintering (SLS) can provide a vast array of products of unique architecture [2]. 

FDM is a quite simple and inexpensive method of producing 3D objects with relatively high resolution. In this case, the geometry of the object is formed by extruding a thermoplastic filament. The quality of the final product depends on various parameters, such as the thickness of the layer, the air gap, the orientation of the structure, the density of the material and others, for example, the operator's experience [2,3]. 

FDM technology is based on the layered design of physical models taken directly from the digital 3D solid model. The printhead locally heats the input material in the form of a plastic rod, causing its local melting. Then, the head moves in two directions, X and Y, and spreads the material layer at the place of the current cross-section of the model building. After finishing the layer, the construction table is lowered by one-layer thickness, and the whole process is repeated until the entire model is finished [4]. However, this process requires polymeric filament with specific physical-chemical properties, especially thermal and rheological, to allow easy processing and, consequently, the manufacturing of products that meet high-performance requirements [2].

Nevertheless, even with the best 3D printing parameters, the strength properties of the final product will be worse in comparison to those offered by other methods. The solution to this issue may consist either in manufacturing composites or in the use of various methods of post-production processing, e.g. crystallization.

Crystallization process can be carried out using heat treatment, which is recommended for elements produced by 3D printing. This process is presented as an effective way to increase the mechanical properties of materials via the optimization of their crystallinity. In the case of semi-crystalline materials, crystallization reduces the mobility of polymer chains and therefore increases the strength. Besides, heat treatment is a method known to reduce internal stress that may be accumulated during the 3D printing process: it can prevent the further spread of structural defects [5,6].

In additive methods, polymers are considered the most useful materials to process because they can be easily adapted to a particular method and their processing temperature is relatively low. Acrylonitrile butadiene styrene (ABS), polylactide (PLA), polycarbonates (PC), polyethylene terephthalate (PET) are therefore widely applied, all their disadvantages notwithstanding. However, these are ABS and PLA that excel in terms of price. ABS has good strength properties; although, during processing, it gives off an unpleasant odor. PLA is considered an environmentally friendly material and is frequently used despite its low strength properties [7].

PLA is an aliphatic thermoplastic polyester that is characterized by high modulus, high strength and good clarity. Therefore, it has aroused great interest as a potential replacement for petroleum-based polymers. Its biocompatibility and bioresorbability make it appropriate in such medical applications as drug delivery systems, seams, blood vessel surgery, etc. However, PLA is used in the household industry, especially in the packaging industry, due to its good mechanical properties, transparency and compostability [8,9,10,11,12,13]. Lactide acid is a naturally occurring organic acid, and its monomers can be made from non-toxic raw materials. Every year, it is produced from renewable resources [14,15,16]. Due to its applicability in the food packaging industry, it has been classified as generally recognized as safe (GRAS) by the United States Food and Drug Administration (FDA). One of the disadvantages of PLA is its glass transition temperature amounting to around 60 °C, which results in the loss of its strength properties at higher temperatures [17,18,19].

Various researchers have studied PLA composites. Among the fillers added to reinforce PLA material are carbon nanotubes, carbon black, graphene, metal powders which improve the electrical and thermal properties as well as organic infills such as wood flour or cellulose fibers used because of their availability, low specific gravity, renewable nature and biodegradability [20,21,22,23,24,25,26].

One of the ways to improve the properties of PLA is the crystallization process. Many researchers made such attempts; for example, in [27,28] it was shown that heat deflection temperature (HDT) and Vicat penetration temperature raised by more than 30 and 100 °C, respectively, after full crystallization of amorphous samples. It also led to an increase in modulus of elasticity, bending strength (by 25%) and impact resistance. Furthermore, the rate of enzymatic degradation can be reduced by more than seven times for highly crystalline PLA as compared to its amorphous samples [29]. 

Drieskens et al. showed that, in the case of crystallized PLA oxygen and water vapor, permeability coefficients decreased more than 4 and 3 times, respectively, compared to the amorphous referential samples [30]. Everything mentioned above underlines the importance of PLA crystallization not only from the practical point of view but also for obvious reasons related to market development.

The crystallinity of polymers is also influenced by different types of applied nucleating agents. The impact of six nucleating agents (NA), i.e. orotic acid (OA); potassium 3,5-bis (methoxycarbonyl) benzenesulfonate (LAK-301); aryl-substituted phosphate salt (TMP-5); talc (TALC); N′1, N′-6-dibenzoyl-adipohydrazide (TMC-306) and N1, N1′-(ethane-1,2-diyl) bis (N2-phenyloxalamide) (OXA), on the behavior of PLA in crystallization were examined by Feng Y. et al. by comparing the DSC results. The results showed that, depending on the additives, crystallization temperature might be lowered and crystallization time might be reduced by half, which is very important for the rapid production of highly crystallized PLA materials [10,31]. The aim of this study was to evaluate changes in physical-mechanical properties of the samples manufactured by 3D printing technology using PLA that is subsequently subjected to crystallization. Such studies have, of course, already been discussed by other researchers who indicated the positive effect of PLA crystallization on mechanical properties [5,6,32,33,34]. Feng also discusses the impact of water absorption by the samples produced by the 3D method on their mechanical properties (which are known to be sensitive to water ageing). Additionally, the results of the tests of the impact of low-cycle loads on the properties of the samples produced by the 3D method are presented; as far as we know, such an approach has not been widely adopted yet. Interestingly enough, the results showed that the energy dissipation effect in the researched material was largely unaffected by water aging, which is visible in composite materials. 

Bher et al. showed that insertion of GRH oxide into starch oxide and matrix of PLA increases the mechanical and barrier properties of tested material. Interposition the filling into commonly known materials is a widely used practice by modern scientists [35]. A common concept of increasing the properties of polymeric materials is modifying their chemical composition with appropriate additives. One of the methods for improving the properties of materials is to add graphene particles to their structure. Sanes et al. proved that this is a very promising method in modern engineering. Great importance is attached to biodegradable and biocompatible nanocomposites. Some promising knowledge is that the hydrolytic degradation of PLA in NaOH solution was faster with PLA nanocomposites with nanophase content than with pure PLA. There are also many other materials used to make nanocomposites. For many years, PP was one of the most known material used for modification with graphene [36,37]. 

## 2. Materials and Methods 

### 2.1. Materials

In this work, samples of neat polylactic acid filament (Nature Works) were investigated. Samples were made in 4 variants, which depended on the degree of infill. The 3D printing parameters are presented in Table 1. Then, half of the samples labelled with the letter “C” were crystallized at 70 °C for 1 h and subsequently subjected to slow air cooling for 24 h. Filaments of 1.75 mm diameter were produced by Spectrum Company (Warszawa, Poland). The samples were made on 3D printer Prusa i3 MK3S 1.75 mm in by FDM method. In 2012, Josef Prusa invented Prusa i3 MK2 [38]. This 3D printer was made in the Czech Republic, and in 2016 it was the most used 3D printer in the world [39,40]. The samples were printed in shape typical of the standard samples for strength tests in accordance with the standard EN ISO 3167. Garzon-Hernandez et al. showed that layer heights have great importance in modeling material properties. It is associated with the occurrence of voids between the layers of the material, which directly affects the strength properties [41].

### 2.2. Method of testing

In this study, samples were produced from polylactic acid filament Spectrum at the Cracow University of Technology (Cracow, Poland). Static tensile test (PN-EN ISO 527-1:20100), bending test (PN-EN ISO 178:2011) and hysteresis loops were conducted with an MTS Criterion Model 43 machine (MTS System Corp., Eden Prairie, MN, USA) with speed 2 mm/min. In order to perform a more precise measurement, an extensometer was used for mechanical tests. Impact strength (PN-EN ISO 179-1:2010) was measured with Zwick HIT 5.5P (Zwick Roell Group, Ulm, Germany). Softening point (PN-EN ISO 306) was measured with Ceast series 6506 (Torino, Italy). The temperature rise step was set up to 50 °C per 1 h. SEM images were taken with a scanning electron microscope at 1 × 35 and 1 × 100 magnification. Density measurements were conducted with RADWAG WAS 22W (Radom, Poland). Thermal analysis by differential scanning calorimetry (DSC) was performed using an STA 409 CD Netzsch apparatus. The specimens for the DSC test were prepared in the same way as the samples for mechanical characterization. All the samples, about 10 mg weight each, were cut from the original ones. The heating scan embraced the range from 30 to 200 °C with the heating rate of 10 °C/min under nitrogen atmosphere to prevent polymer degradation.

Hydrolytic degradation was performed in distilled water with the addition of NaCl (2 wt.%), imitating physiological saline container at 40 °C, for 1 d, 4 weeks and 8 weeks, and calculated according to the following equation:

(1) $$M\%=\frac{M_{w}-M_{t}}{M_{t}}\times 100\%$$

*M* − water uptake
*M*_w_ − mass of the samples after hydrolytic degradation
*M*_t_ − mass of the neat samples before hydrolytic degradation

In order to determine the effect of hydrolytic degradation, mechanical tests were performed after 4 and 8 weeks of soaking in saline solution.

## 3. Results and discussion

Table 2 shows the density results for different degree of polylactide (PLA) infill. As the percent of added infill increases, the density also does. The samples subjected to the crystallization process were characterized by higher density, which results from the fact that, during crystallization, the material shrinks and the reduction in volume for the same mass takes place. The average of the PLA density was determined to be 1.25–1.27 g/cm^3^. The obtained results indicate that the difference in density for the samples with 100% of infill addition did not exceed 3% as compared to the others.

One of the advantages of polymer composites is their high specific properties. The specific strength was calculated as the ratio of the tensile strength and density and is presented in Table 2. It shows that with the decreasing amount of infill, the density and strength decrease, but their ratio hardly changes. The reduction of the amount of infilling had positive impact on the crystallization behavior. The specific strength increases after thermal treatment for the composites filled in 85 and 70% was 15 and 18%, respectively, where for 100% infill—12%. The lowest differences were noted for the lowest degree of filling. This is most likely due to the insufficient load bearing capacity of composites with only 50% fill. Crystallization also contributed to the reduction of differences in relation to 100% infilling. Specific strength decreased for non-crystallized 85% by 17%, while after crystallization by 14%, in the case of composites filled in 70%, the decrease before crystallization was 15% and after crystallization was 11%.

Table 3 compares the strength properties of PLA for decreasing degree of infill determined in tensile and three-point bending test applied to the crystallized samples not subjected to thermal treatment. As the degree of infill decreases, the tensile strength decreases by about 20%. There is a significant difference in strength between 100% and 50% – filled samples. The strength drops from the level of 64 MPa to 43 MPa. However, despite the reduction of the content of the material by half, the tensile strength decreased by about 30%. The tensile strength value of 64 MPa for the samples with 100% of infill addition corresponds with the value of strength produced by injected samples. The modulus of elasticity changes as tensile strength does. Bending strength is twice as high compared to the tensile strength, which is related to the method of carrying out the test, the three-point distribution of bending stresses, but also to the method of producing single filament "threads" using the FDM method. The crystallization process of the material results in the increase in strength and modulus both in tension and bending test. Brischetto et al. showed interesting research. The honeycomb structure was produced inside the samples made of PLA and ABS. Then, it was subjected to bending tests. This structure can keep the mechanical properties at the same level by reducing the weight of the component [42]. This was shown by Ferro et al. This can be an extremely useful technique during constructing drones. Authors of this publication draw attention to the fact that, by using numerical and static mathematical analyzes, we can design a process with maximum elimination of errors. This will most likely be the subject of further research on other materials [43].

The deformation of tensile-tested samples is lower by about 50–70% in relation to the samples subjected to the bending test. Brischetto et al. showed that specimens subjected to the tensile test achieve results at a level very similar to the results obtained in the above studies. The results obtained by researchers were compared for samples produced only with 100% infill [44].

**Figure 1:**
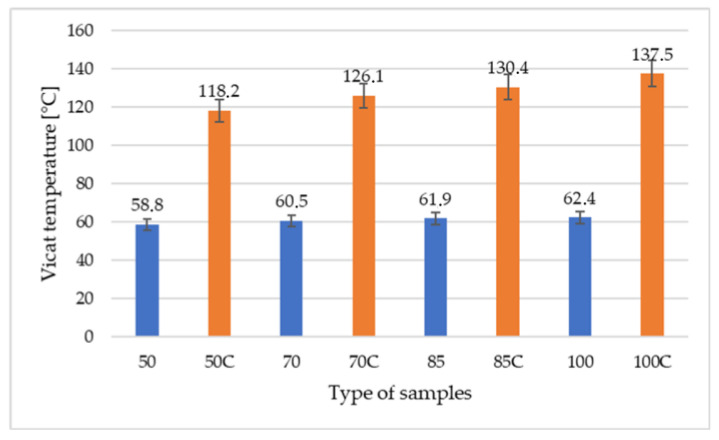
Comparison of the impact of the percent of added infill on Vicat Temperature.

Results presented in Figure 1 show the influence of the infilling content and thermal treatment on the Vicat softening temperature (VST). As can be seen, the differences in infilling had no significant effect on the VST value. The highest differences were noted for the lowest degree of filling (50%), the decrease was 6 and 14% for untreated and treated, respectively (compare to 100% infill). The reason for this decrease was the increase in empty spaces. It is worth noting that the thermal treatment of PLA composites significantly contributed to the increase in VST. The highest differences were obtained for 100% infilling—120%—and the smallest for 50% filling—101%. With less material, there is less space for crystal nucleation.

**Figure 2:**
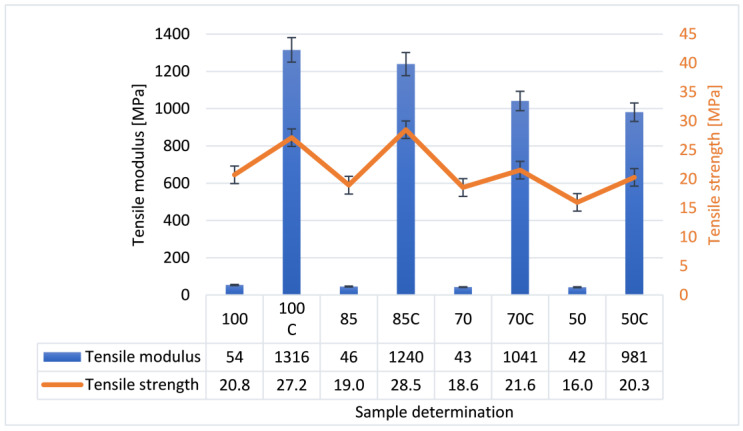
Comparison of mechanical properties examined during static tensile test for untreated and crystallized samples measured at 70 °C.

Figure 2 presents the test results for composites obtained at elevated temperature which caused a significant decrease in their value. In the case of PLA, it is assumed that the melting point occurs at 180 °C and the glass transition temperature—at 60 °C [45].

**Figure 3:**
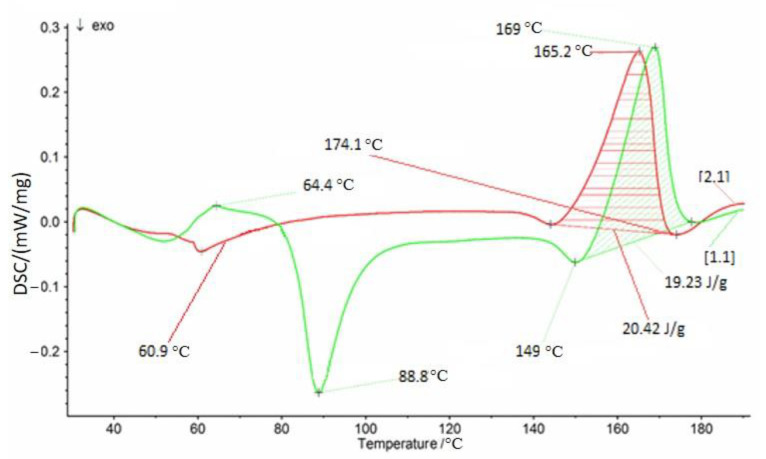
DSC thermal curves for pure PLA (green curve) and PLA after crystallization process (red curve).

The key element that influences the mechanical and physical properties of PLA is the morphology, and form and degree of crystallinity. This happens because PLA can take an amorphous or semi-crystalline form [46]. Heat treatment and the PLA crystallization process result in an increase in the elasticity modulus by several dozen times and in a 30% increase in tensile strength. As in the case of tests conducted at ambient temperature, strength properties of PLA produced by the 3D printing method decreased with the decreasing percent of infill. To confirm that observation, DSC measurement was carried out for PLA with 100% of added infill. Figure 3 presents DSC curves registered during the test for pure PLA (green line) and PLA after crystallization (red line).

The green curve represents a PLA sample not subjected to an annealing process. The sample crystallized during the heating process melted and changed its structure. Cold crystallization started immediately after the glass transition point was crossed, and the mobility of the chains increased. The red curve recorded for the sample heated at 70 °C for 1 h does not show cold crystallization, and the melting enthalpy was higher, which indicates a higher degree of crystallinity.

Based on the above data, the degree of crystallinity was calculated from the formula below:

(2) $$X_{c}=\frac{\Delta H_{f}}{\Delta H_{f}^{0}\;}\;\times 100\%\;$$

Δ*H_f_* is the melting enthalpy of the samples
$\Delta H_{f}^{0}$ is the melting enthalpy of 100*% crystalline* polymer, which for *PLA* is equal to 110 *J*/*g* [39].

On the basis of the above-mentioned results contained in the Table 4, it is noticeable that the samples not subjected to the crystallization process have a lower degree of crystallinity in relation to the samples subjected to crystallization. A higher degree of crystallization results in higher mechanical properties, this relationship was also confirmed in this study.

Another very important feature of polymeric materials is their water absorption and changes in properties triggered by the ageing process, which may include, e.g., self-stress relaxation, recrystallization, phase dispersion in multicomponent systems, softener migration, the formation of stress and fatigue cracks, thermo-oxidative degradation, or evaporation or migration of volatile components [47,48]. All these effects affect mechanical properties.

**Figure 4:**
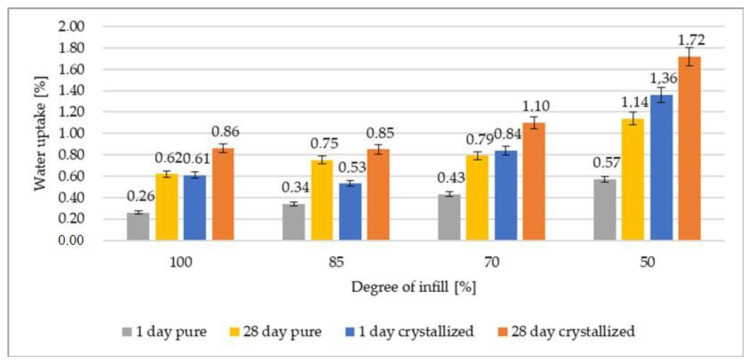
Weight gain resulting from hydrolytic degradation after soaking pure and crystallized samples for 1 and 28 d.

Figure 4 shows the effect of hydrolytic degradation on pure and crystallized samples with varying percent of added infill after 1 and 28 d of soaking. In the case of the crystallized samples, water absorption was much higher than in the untreated samples. However, the difference between water absorption of the samples tested after 1 and 28 d was smaller in the case of crystallized samples in comparison to the pure ones. The smaller degree of infill causes that material to have more free spaces into which water can penetrate. 3D-printed PLA is hygroscopic even with the 100% addition of infill. On the first day of water sorption, the samples after crystallization had a much higher water absorption capacity than the samples before thermal treatment. The initial significant water uptake by the crystallized samples resulted in smaller uptake variations after 28 d. On the other hand, with the reduction of the infilling degree of composites, their water absorption capacity increased. The lower the filling, the higher the water uptake capacity (85% infill—31% increase, and 50% infill—120% increase after 1 d in relation to 100% infilling for samples without heat treatment). Solomon et al. showed that parameters selected for the printing process are very important. As mentioned in that article, the degree of infill results in a property change. Therefore, it is extremely important to carefully consider the selection of the degree of infill [49].

**Figure 5:**
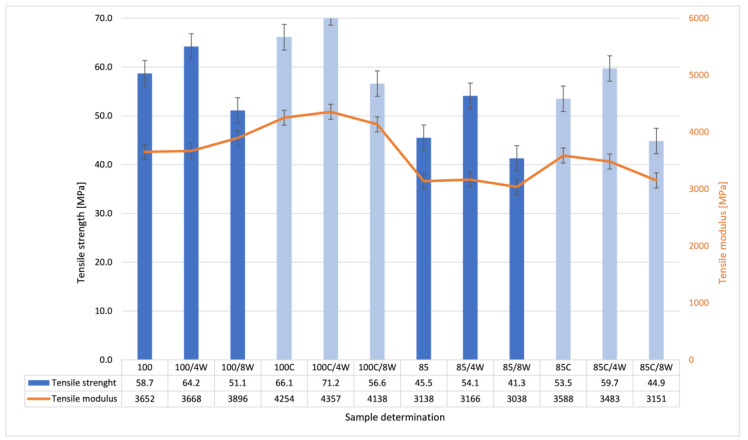
Comparison of mechanical properties examined during static tensile tests conducted at room temperature for pure, hydrolytically degraded and crystallized samples.

**Figure 6:**
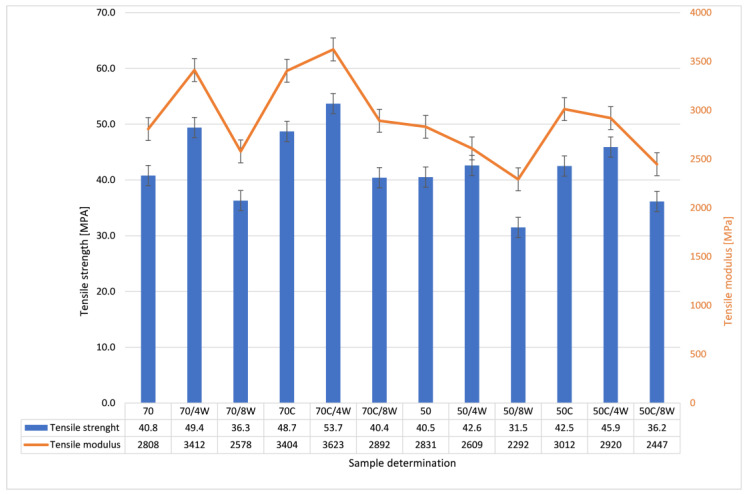
Comparison of mechanical properties examined during static tensile tests conducted at room temperature for pure, hydrolytically biodegraded and crystallized samples.

As the printing process is fraught with errors, the production of seemingly solid elements does not guarantee their perfect fusion. Figure 5 and Figure 6 present the comparison of mechanical properties examined during static tensile tests conducted at room temperature for neat, hydrolytically degraded and crystallized samples. A clear increase in strength and modulus of elasticity was observed for the materials after crystallization, especially for those with the higher percentage of added infill (100, 85 and 70%). The effects of hydrolytic degradation were clearly visible after 8 weeks of soaking in physiological saline. Crystallized samples maintained high strength properties even after 8 weeks of hydrolytic degradation.

**Figure 7:**
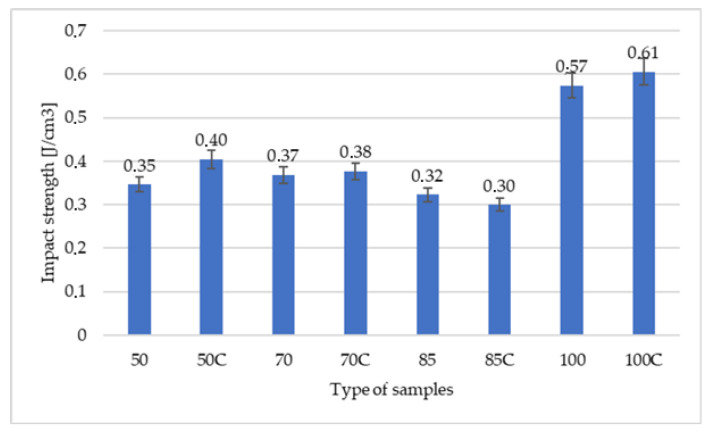
Impact strength depending on the percent of added infill and crystallinity of samples.

Figure 7 compares the relationship between the impact strength and the percent of added infill and crystallinity of samples. Two effects can be observed here; namely, the crystallization slightly increases the impact resistance (despite a slight decrease in deformation at break), and the failure work is greater due to the greater maximum force. The highest value of impact toughness was measured in the samples with 100% of added infill. The impact strength decreased by 30–40% with 85% addition of infill, which was probably caused by the shape of cavities in the matrix.

The interesting idea is to test the analyzed materials during the first load cycles. Potential failure mechanisms in such materials may be indicated and elucidated by the thorough examination of the relationship of stresses and strains during their stretching. In the case of solid plastics, the classical theory of elasticity is applied to small and short-term stresses: only one strain value corresponds to each stress value. However, due to the viscoelastic nature of plastics during deformation, stresses and strains do not occur in phase and, consequently, the appearance of a hysteresis loop can be observed. The area of the obtained hysteresis loop represents the energy dispersed in one complete cycle. There is the difference between the energy converted into heat, the elastic potential energy under load, and the energy obtained in return during unloading.

Depending on the value and method, changes occurring with an increase in the number of cycles have different impacts on the material [50]. If the sample is subjected to symmetrical cycles of deformation, one can observe three effects: increase in maximum stress, which indicates cyclical reinforcement of the material; decrease in maximum stress with the number of cycles until saturation state that stands for the cyclical weakening of the material or, last but not least, no effects whose lack means that the material is in a stable state.

**Figure 8:**
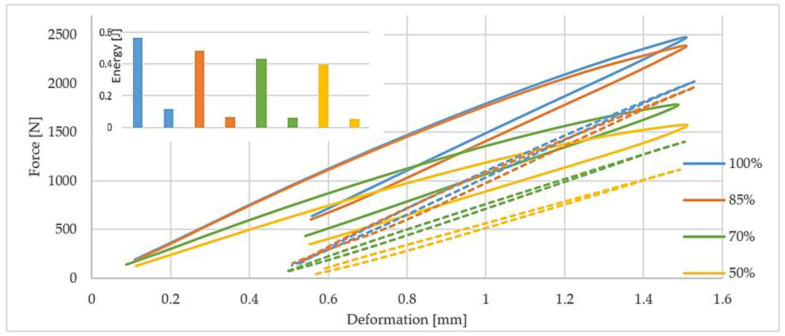
Influence of the percent of infill added to the samples produced by 3D printing on the shape of the hysteresis loops obtained after 1^st^ and 50^th^ load cycles Solid line—first loop, dashed line—fiftieth line.

Figure 8 shows the effects of mechanical energy dissipation hysteresis loops registered after the first and fiftieth load cycles and changes in the dissipated energy between these cycles. As the percent of added infill decreases, the ability to discharge mechanical energy decreases proportionally for 1 and 50 loops. The slope of the loop decreases with the drop of the percentage of the added infill and with the progress of the fatigue process. The decrease in the percentage of added infill is accompanied by a drop in the value of the modulus of elasticity, which was confirmed by static tests and progressive cyclic load tests. It was due to material heating and a friction of individual fibers bundled one with another.

**Figure 9:**
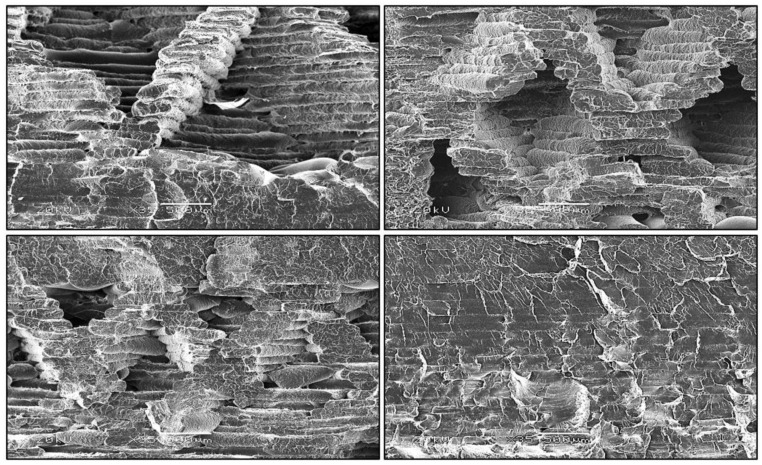
Stretch fracture images showing the changes triggered by the successive increase in the percent of added infill on SEM images at 35 × magnification.

One of the main disadvantages of the elements produced by the 3D printing method is their porous structure. The porosity of the material can be high and cause a significant decrease in strength properties due to, among others, the limitation of the internal bond between the layers, which is presented in Figure 8. The pore formation is largely dependent on the selected 3D printing method. In the case of FDM, pores are relatively large, being thus considered as structural defects that can lead to delamination between the layers of the material. High porosity, however, can be an advantage if the material is meant to be a foamed one; it all depends on its intended use [7]. Microscopic observations were made to determine the structure of the produced samples. Figure 9 shows that observable changes occur in the filament arrangement with the increase in the percent of infill in the samples. As the infill level increases, the orientation of the filament bundles changes, which is related to their stretching and increase in their thickness. The amount of infill alone, even at the optimal angle of 45°, does not cause deformation of the resulting bundles and gluing of individual filaments.

**Figure 10:**
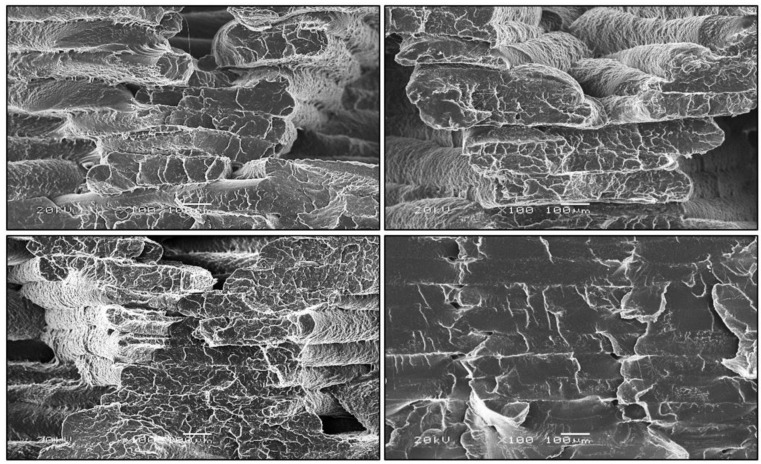
Stretch fracture images for the successive increase in the percent of added infill on SEM images at 100 × magnification.

In Figure 10, at 100 × magnification, the geometries of individual filaments fused together in bundles are noticeable. Growing addition of infill results in the more regular shape of the single filament thread. A smaller addition of infill promotes the deformation of both a single thread and a whole bundle of filaments. The thickness of a single filament bundle at 50% addition of infill ranges from 60 to 160 μm and is parallel to each other and decreases to 60–80 μm at 100% addition of infill.

**Figure 11:**
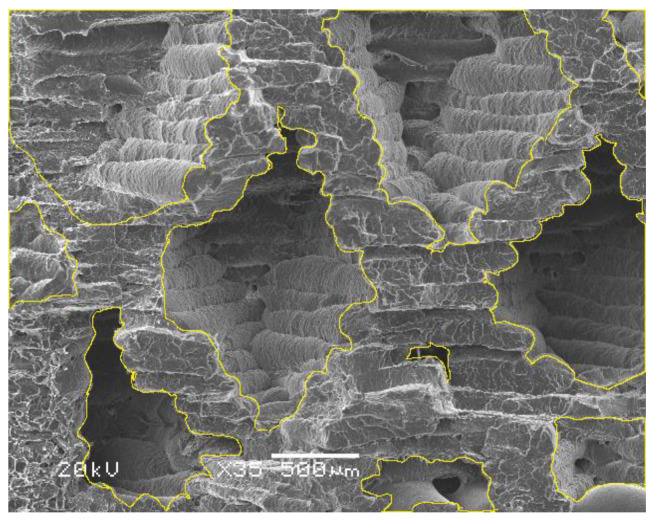
Exemplary SEM image showing how to calculate the real amount of infill in the image using the ImageJ program.

The percentage of empty spaces was also checked, as is in shown in Figure 11. SEM images were analyzed using ImageJ software to measure the actual amount of infill in each of them.

Figure 11 shows how to measure and sum up the empty spaces in the given sample. It presents the average results of the examination, made on the basis of the analysis of 5 SEM images each of which reflected the particular percent addition of infill to PLA. Garzon-Hernandez et al. showed that empty spaces between the layers have great importance on mechanical properties on subjected samples. When modeling the properties of samples, considerable attention should be paid to the height of the material printed by the FDM method. In the future, this will result in changes in the values obtained during mechanical tests [41,51].

To determine the circularity of empty spaces in the samples made of PLA, the shape factor was calculated using the Feret method. The smallest and largest diameters of voids were measured and calculated according to the following equation:

(3) $$R=\frac{F_{\min}}{F_{\max}}$$

*F*_max_ − maximum length of empty space
*F*_min_ − minimum length of empty space

**Figure 12:**
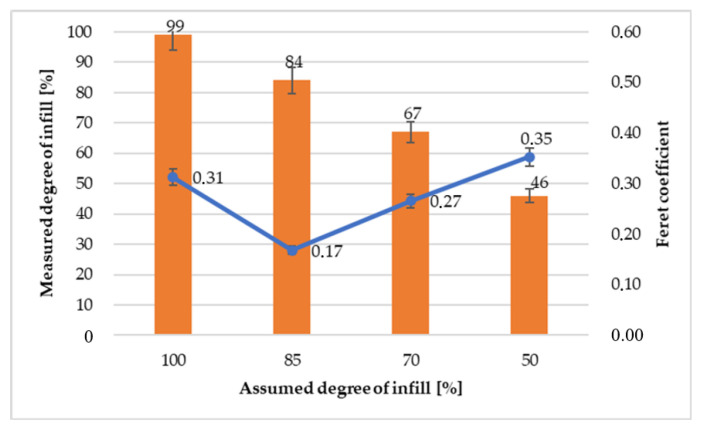
Comparison of the real and assumed amount of infill and Feret coefficient.

When calculating the Feret coefficient, it is assumed that the ratio of the minimum to the maximum diameter for a circle is equal to one. Therefore, the lower result of the Feret coefficient suggests that the voids are more spherical in cross-section. A noticeable relationship can be observed in Figure 12, showing that the samples with 100% addition of infill have the shape closer to the circle. This may be due to the fact that almost uniform filling of the sample reduces the likelihood of uneven structures. However, such circularity is similar to the shapes of empty spaces in the samples with 50% addition of infill. Most likely, it stems from the fact that a much larger number of voids does not generate the risk of excessive deformation of individual fiber particles. Figure 12 shows slight differences between the assumed addition of infill and its real value.

## 4. Conclusions

The modern world is increasingly focused on recycling. During chemical depolymerization or hydrolysis, it is possible to recycle polylactic acid into its monomers. By purifying PLA monomers, it is possible to recycle the material without losing its original properties. PLA can be used in a variety of products. It is a biodegradable polymer that uses carbon dioxide to carry out the reaction during the manufacturing process. These are very important factors that make it necessary to focus on this polymer during engineering design. In the case of biodegradation, PLA can be subjected to industrial composting, which makes PLA degradable in 100% [52,53,54]. Hydrolytic degradation is the basis for the recycling. In this study, we showed that weight loss in the aquatic environment reduces the weight of PLA, which results in slow degradation. Image analysis methods were used to verify the degree of filling, thereby making it possible to determine the actual degree of filling.

This study evaluates changes in physical-mechanical properties of PLA samples manufactured by 3D printing technology. The percent addition of infill has a significant impact on the mechanical properties of materials made by 3D printing. Most likely, this results from the fact that empty spaces between the individual filament threads favour the formation of cracks and increase the rate of material degradation. With the increasing percentage of infill addition, the value of mechanical energy dissipation increases, which results in the possibility of greater relaxation of internal stresses. Samples were also subjected to the crystallization process, and they proved to have better mechanical properties, which may be related to the sticking of fibers due to crystallization shrinkage, which also slightly reduces the strain at break. The hydrolytic degradation examination has0020shown that the percentage of infill addition has a significant impact on water absorption. This happens because the lower the amount of infill, the more internal gaps occur in the material that potentially affect the ingress of water into the sample. Besides, knowledge of the properties of PLA at various percent addition of infill allows for better design of materials in terms of strength and environmental requirements. Additionally, such a comprehensive analysis, taking into account the influence of the infill level, hydrolytic degradation on the mechanical properties, may be used in the future for the design of composites manufactured by 3D printing.

## Figures and Tables

**Table 1:** Parameters of 3D printing.

Parameters	Variant 1	Variant 2	Variant 3	Variant 4
Layer height [mm]	0.5			
Bed temperature [°C]	55			
Extruder temperature [°C]	190			
Extrusion speed [mm/s]	45			
Degree of infill [%]	100			
Direction of print angle [°]	45	85	70	50

**Table 2:** Description of the tested materials (density and specyfic strength).

Index	Description	Density [g/cm^3^]	Specific Strength
100	100% of infill	1.2156 ± 0.0321	48.3 ± 0.4
100C	100% of crystallized infill	1.2253 ± 0.0222	53.9 ± 0.6
85	85% of infill	1.1652 ± 0.0139	40.0 ± 0.2
85C	85% of crystallized infill	1.1655 ± 0.0092	45.9 ± 0.7
70	70% of infill	0.9946 ± 0.0139	41.0 ± 0.6
70C	70% of crystallized infill	1.0101 ± 0.0065	48.2 ± 0.5
50	50% of infill	0.8704 ± 0.0294	46.5 ± 0.8
50C	50% of crystallized infill	0.8767± 0.0118	48.5 ± 0.3

**Table 3:** Comparison of strength properties and Young modulus in neat and crystallized samples subjected to stretching and bending tests, P-Pure samples, C-crystallized samples.

	σ_z_ [MPa]		E_z_[MPa]		ε_z_[%]		σ_g_ [MPa]		E_g_[MPa]		ε_g_[%]	
	P	C	P	C	P	C	P	C	P	C	P	C
PLA 100	64.2±0.4	66.1±0.3	3668±121	4254±210	2.2±0.2	1.9±0.3	118.9±0.4	119.1±0.3	3487±78	3526±131	5.2±0.5	7.4±0.2
PLA 85	54.1±0.2	53.5±0.4	3466±132	3588±195	2.5±0.3	2.1±0.5	102.5±0.3	101.5±0.2	3279±104	3305±101	6.1±0.4	5.9±0.3
PLA 70	49.4±0.4	48.7±0.5	3412±129	3404±187	2.1±0.5	2.0±0.2	95.7±0.1	95.6±0.6	3138±109	3096±89	5.2±0.3	5.0±0.2
PLA 50	42.6±0.3	42.5±0.6	2609±147	2920±217	2.4±0.4	2.2±0.6	89.8±0.4	81.2±0.2	2903±119	2980±108	5.6±0.1	5.5±0.2

Footnotes: σ_z_ − tensile strength, E_z_ − modulus of elasticity, ε_z_ − strain at break, σ_g_ − bending strength E_g_ − bending modulus, ε_g_ − strain at break bending.

**Table 4:** Comparison of the crystallinity degree of samples before and after the crystallization process.

Types of Samples	Samples before Crystallization	Samples after Crystallization
Values of melting enthalpy [J/g]	19.23	20.42
Values of crystallinity [%]	17.48	18.57
